# Supplementary material for: Distribution of antibiotic resistance genes and antibiotic residues in drinking water production facilities: Links to bacterial community
Source: PLoS One. 2024 May 23;19(5):e0299247. doi: 10.1371/journal.pone.0299247 (PMC11115235; doi:10.1371/journal.pone.0299247)
Supplement: S1 Table — F- Forward primer and R- Reverse primer. (DOCX) [file pone.0299247.s001.docx]

**S1 Table: Oligonucleotide primers for the end-point PCR amplification of *ampC, bla_TEM._ IntI1, ermB, ermF, Sul1* and *Sul2* genes. F- Forward primer and R- Reverse primer.**

| **Target gene** | **Primer's name** | **Sequence (5'…..3')** | **Size**  **(bp)** | **PCR conditions** | **References** |
| --- | --- | --- | --- | --- | --- |
| *ampC* | *AmpC*-F | TTC TAT CAA MAC TGG CAR CC | 550 | 94ºC for 5 min, 35 cycles of 94ºC for 5 min, 49ºC for 30 s, 72ºC for 1 min and 72 ºC for 7 min | [1] |
|  | *AmpC*-R | CCY TTT TAT GTA CCC AYG A |  |  |  |
| *bla_TEM_* | TEM-F | ATT CTT GAA GAC GAA AGG GC | 1 150 | 94ºC for 5 min, 30 cycles of 95ºC for 5 min, 60ºC for 1 min, 72ºC for 1 min and 72 ºC for 5 min | [2] |
|  | TEM-R | ACG CTC AGT GGA ACG AAA AC |  |  |  |
| *IntI1* | HS463A | CTG GAT TTC GAT CAC GGC ACG | 473 | 94ºC for 5 min, 30 cycles of 94ºC for 30 s, 64ºC for 30 s, 72ºC for 1 min and 72 ºC for 5 min | [3] |
|  | HS464 | ACA TGC GTG TAA ATC ATC GTC G |  |  |  |
| *ermB* | *ermB*-F | GAA AAG GTA CTC AAC CAA ATA | 638 | 95ºC for 5 min, 35 cycles of 95ºC for 30 s, 48 ºC for 1 min, 72ºC for 2 min and 72 ºC for 10 min | [4] |
|  | *ermB*-R | AGT AAC GGT ACT TAA ATT GTT TAC |  |  |  |
| *ermF* | *ermF*1 | CGG GTC AGC ACT TTA CTA TTG | 466 | 95ºC for 5 min, 35 cycles of 95ºC for 30 s, 50ºC for 30 s, 72ºC for 2 min and 72 ºC for 10 min | [5] |
|  | *ermF*2 | GGA CCT ACC TCA TAG ACA AG |  |  |  |
| *Sul1* | SulI-F | TTC GGC ATT CTG AAT CTCAC | 822 | 94ºC for 3 min, 35 cycles of 94ºC for 1 min, 60ºC for 1 min, 72ºC for 1 min and 72 ºC for 5 min | [6] |
|  | SulI-R | ATG ATC TAA CCC TCG GTC TC |  |  |  |
| *Sul2* | SulII-F | CGG CAT CGT CAA CAT AAC C | 722 | 94ºC for 3 min, 35 cycles of 94ºC for 1 min, 60ºC for 1 min, 72ºC for 1 min and 72 ºC for 5 min | [7] |
|  | SulII-R | GTG TGC GGA TGA AGT CAG |  |  |  |

**References**

1. Coertze RD, Bezuidenhout CC. The prevalence and diversity of AmpC β-lactamase genes in plasmids from aquatic systems. Water Science and Technology. 2018;2017(2):603-11.

2. Costa D, Poeta P, Sáenz Y, Coelho AC, Matos M, Vinué L, Rodrigues J, Torres C. Prevalence of antimicrobial resistance and resistance genes in faecal Escherichia coli isolates recovered from healthy pets. Veterinary microbiology. 2008;127(1-2):97-105.

3. Labbate M, Chowdhury PR, Stokes HW. A class 1 integron present in a human commensal has a hybrid transposition module compared to Tn *402:* evidence of interaction with mobile DNA from natural environments. Journal of bacteriology. 2008;190(15):5318-27.

4. Tran CM, Tanaka K, Watanabe K. PCR-based detection of resistance genes in anaerobic bacteria isolated from intra-abdominal infections. Journal of infection and chemotherapy. 2013;19(2):279-90.

5. Chung WO, Werckenthin C, Schwarz S, Roberts MC. Host range of the *ermF* rRNA methylase gene in bacteria of human and animal origin. Journal of Antimicrobial Chemotherapy. 1999;43(1):5-14.

6. Chaturvedi P, Singh A, Chowdhary P, Pandey A, Gupta P. Occurrence of emerging sulfonamide resistance (*sul1* and *sul2*) associated with mobile integrons-integrase (*intI1* and *intI2*) in riverine systems. Science of The Total Environment. 2021;751:142217.

7. Pérez-Pérez FJ, Hanson ND. Detection of plasmid-mediated AmpC β-lactamase genes in clinical isolates by using multiplex PCR. Journal of clinical microbiology. 2002;40(6):2153-62.
