# Supplementary material for: Distribution of antibiotic resistance genes and antibiotic residues in drinking water production facilities: Links to bacterial community
Source: PLoS One. 2024 May 23;19(5):e0299247. doi: 10.1371/journal.pone.0299247 (PMC11115235; doi:10.1371/journal.pone.0299247)
Supplement: S2 Table — (DOCX) [file pone.0299247.s002.docx]

**S2 Table: Primers: Oligonucleotide primers for the end-point PCR amplification of six pAmpCs, namely ACC, EBC, DHA, FOX, CIT and MOX gene: F- Forward primer and R- Reverse primer**.

| **Target gene** | **Primer's name** | **Sequence (5'…..3')** | **Size**  **(bp)** | **PCR conditions** | **References** |
| --- | --- | --- | --- | --- | --- |
| ACC | ACCMF | AACAGCCTCAGCAGCCGGTTA | 346 | 94ºC for 3 min, 35 cycles of 94ºC for 30 s, 64ºC for 30 s, 72ºC for 1 min and 72 ºC for 7 min | [1] |
|  | ACCMR | TTCGCCGCAATCATCCCTAGC |  |  |  |
| MIR-1T ACT-1 | EBCMF | TCGGTAAAGCCGATGTTGCGG | 302 | 94ºC for 3 min, 35 cycles of 94ºC for 30 s,64ºC for 30 s, 72ºC for 1 min and 72 ºC for 7 min |  |
|  | EBCMR | CTTCCACTGCGGCTGCCAGTT |  |  |  |
| FOX-1 to FOX-5b | FOXMF | AACATGGGGTATCAGGGAGATG | 190 | 94ºC for 3 min, 35 cycles of 94ºC for 30 s, 64ºC for 30 s, 72ºC for 1 min and 72 ºC for 7 min |  |
|  | FOXMR | CAAAGCGCGTAACCGGATTGG |  |  |  |
| DHA-1, DHA-2 | DHAMF | AACTTTCACAGGTGTGCTGGGT | 405 | 94ºC for 3 min, 35 cycles of 94ºC for 30 s, 64ºC for 30 s, 72ºC for 1 min and 72 ºC for 7 min |  |
|  | DHAMR | CCGTACGCATACTGGCTTTGC |  |  |  |
| LAT-1 to LAT-4, CMY-2 to CMY-7, BIL-1 | CITMF | TGGCCAGAACTGACAGGCAAA | 462 | 94ºC for 3 min,35 cycles of 94ºC for 30 s, 62ºC for 30 s, 72ºC for 1 min and 72 ºC for 7 min |  |
|  | CITMR | TTTCTCCTGAACGTGGCTGGC |  |  |  |
| MOX-1, MOX-2, CMY-1, CMY-8 | MOXMF | GCTGCTCAAGGAGCACAGGAT | 520 | 94ºC for 3 min, 35 cycles of 94ºC for 30 s, 59ºC for 30 s, 72ºC for 1 min and 72 ºC for 7 min |  |
|  | MOXMR | CACATTGACATAGGTGTGGTGC |  |  |  |

**References**

1. Pérez-Pérez FJ, Hanson ND. Detection of plasmid-mediated AmpC β-lactamase genes in clinical isolates by using multiplex PCR. Journal of clinical microbiology. 2002;40(6):2153-62.
