# Supplementary material for: Distribution of antibiotic resistance genes and antibiotic residues in drinking water production facilities: Links to bacterial community
Source: PLoS One. 2024 May 23;19(5):e0299247. doi: 10.1371/journal.pone.0299247 (PMC11115235; doi:10.1371/journal.pone.0299247)
Supplement: S3 Table — (DOCX) [file pone.0299247.s003.docx]

**S3 Table: Oligonucleotide primers for the real-time PCR quantification of 16S rRNA, *sul1, ermB* and *IntI1*: F- Forward primer and R- Reverse primer.**

| **Target gene** | **Primer's name** | **Sequence (5'…..3')** | **PCR conditions** | **References** |
| --- | --- | --- | --- | --- |
| 16S rRNA | 906F | AAA CTC AAA KGA ATT GAC GG | hold stage of 50ºC for 2 minutes and 95ºC for 5 minutes, 40 cycles of 95ºC for 15 seconds, 55ºC for 15 seconds and 72ºC for 15 seconds | [80] |
|  | 1062R | CTC ACR RCA CGA GCT GAC |  |  |
| *sul1* | FW | CGCACCGGAAACATCGCTGCAC | hold stage of 50ºC for 2 minutes and 95ºC for 15 minutes, 40 cycles of 95ºC for 15 seconds, 60ºC for 30 seconds and 72ºC for 30 seconds | [26] |
|  | RV | TGAAGTTCCGCCGCAAGGCTCG |  |  |
| *IntI1* | FW | GGCTTCGTGATGCCTGCTT | hold stage of 50ºC for 2 minutes and 95ºC for 15 minutes, 40 cycles of 95ºC for 15 seconds, 55ºC for 30 seconds and 72ºC for 30 seconds | [26] |
|  | RV | CATTCCTGGCCGTGGTTCT |  |  |
