# Supplementary material for: Distribution of antibiotic resistance genes and antibiotic residues in drinking water production facilities: Links to bacterial community
Source: PLoS One. 2024 May 23;19(5):e0299247. doi: 10.1371/journal.pone.0299247 (PMC11115235; doi:10.1371/journal.pone.0299247)
Supplement: Attachment — Submitted filename: Reviewer Report-ARGs & Antibiotic Residues.pdf [file pone.0299247.s005.pdf]

Reviewer Report

The topic of the research is crucial to know the real impact of antimicrobial agents use and which factors contribute to their dispersal and increase ARGs transfer. Therefore, congratulations for carrying out this study because it is important to improve the monitoring of antibiotics and microorganisms in drinking water.

General comments

Most of the manuscript is well-written in a comprehensive way; nonetheless, it is recommended that redaction could be improved for avoiding redundancy.

Consider using “antimicrobial agents” instead when it comes to both antibiotics and fluconazole.

In *Introduction* justify the importance of monitoring fluconazole or antifungals because antibiotics are not the only antimicrobials quantified in this study.

In *Methods and materials* most of the concentration units are represented as fractions (E.g., ml/day), but concentration units of flow rate are not. Please represent all of these similarly.

The *Discussion* section is well explained, and all the results are connected. However, please avoid repeating the data presented in *Results* section.

The title of each section is capitalized but is not the same for the *Results* section. Please use the same format.

Finally, figures should have a good resolution in order to not lose text legibility.

Minor comments

Abstract section

Line 33-34- Please replace “In NW-E, antibiotic and fluconazole concentrations…” with “Regarding antimicrobial agents, antibiotic and fluconazole concentrations…”.

Introduction section

Line 63- It is appropriate to only use the term “human bodies” instead of “human and human bodies” in the same sentence.

Line 79- Please use “antimicrobial agents’ residues” instead of “antibiotic residues because in the study fluconazole is also quantified.

Methods and materials section

Line 95-Please rectify “**environmental DNA (eDNA)**”

Line 128- Verify the version of QIIME2 used in this study because the version of this software contains the year and the month. E.g., QIIME2 Version 2022.4

Line 142- As a suggestion change “for screening for the presence…” by “for screening the presence…”

Line 198- The slope symbol is “m”. Please rectify it.

Line 225-226- It is not clarified in which cases student's t-test and ANOVA were used when data showed a normal distribution.

Results section

Line 274-275- Please detail the temperature results as was made with the other variables. The seasons in which temperature was measured should be specified in Table 2 and Table 3.

Line 299-318- "Unclassified" group should not be mentioned as phyla or genera, so for example in the first case it can be reported that there were six phyla, detailing the proportion from each of them, but a specific percentage of the sequences have not taxonomy assignation.

Discussion section

Line 491-492- Please cite the explanation of nitrite findings.

Line 679-686- An explanation about the risks of fluconazole presence in drinking water is recommended.

Again, congratulations for carrying out this study.
