# Supplementary material for: Distribution of antibiotic resistance genes and antibiotic residues in drinking water production facilities: Links to bacterial community
Source: PLoS One. 2024 May 23;19(5):e0299247. doi: 10.1371/journal.pone.0299247 (PMC11115235; doi:10.1371/journal.pone.0299247)
Supplement: Attachment — Submitted filename: Response to Reviewers.docx [file pone.0299247.s006.docx]

Private Bag X6001, Potchefstroom, 2520, South Africa

Tel:	+27 18 299-1111/2222
Web:	http://www.nwu.ac.za

Tel:	+27 18 299 2379

E-Mail:	24245968@g.nwu.ac.za

11 January 2024

**Academic Editor**

PLOS ONE

Universidad San Francisco de Quito (USFQ)

Campus Cumbayá, Diego de Robles s/n, Quito 170901, Ecuador

# Dear Prof José António Baptista Machado Soares

**SUBMISSION OF REVISED MANUSCRIPT: Distribution of antibiotic resistance genes and antibiotic residues in drinking water production facilities: links to bacterial community**

Please find enclosed our revised manuscript, with the above-mentioned title by Tsholo et al., which we would like to resubmit to *PLOS ONE* after the reviewers’ comments have been addressed. We would like to convey our thanks to both the editor and reviewers for their useful and thorough feedback. Included here are the reviewers’ comments and how the authors have addressed them:

**Prof José António Baptista Machado Soares**

|  | **Comments** | **Response to comments by authors** |
| --- | --- | --- |
| 1 | Please ensure that your manuscript meets PLOS ONE's style requirements, including those for file naming. The PLOS ONE style templates can be found at | Where applicable the style of the manuscripts were revised to match PLOS ONE style templates |
| 2 | In your Methods section, please provide additional information regarding the permits you obtained for the work. Please ensure you have included the full name of the authority that approved the field site access and, if no permits were required, a brief statement explaining why. | There is a memorandum of understanding between North-West University and the local municipality responsible for the two DWPFs. However, the local municipality granted permission for this study on a condition of anonymity (lines 94 to 95). |
| 3 | Please include your amended Funding Statement within your cover letter. We will change the online submission form on your behalf. | The Funding Statement has been amended as following: This work is based on the research supported in part by the National Research Foundation of South Africa Grant No. C2019-2020-00224 (Bursary for Karabo Tsholo), The Water Research Commission (WRC) of South Africa: (Contract – C2019-2020-00224). The views expressed are those of the authors and not of the funding agencies.  The Funding Statement has been included on the cover letter. |
| 4 | We note that the grant information you provided in the ‘Funding Information’ and ‘Financial Disclosure’ sections do not match | The grant information of the Funding Information’ and ‘Financial Disclosure’ now matches. |
| 5 | Please include captions for your Supporting Information files at the end of your manuscript, and update any in-text citations to match accordingly. | Captions of the Supporting Information files were included at the end of manuscript as following; S1 Table, S2 Table, S3 Table, S1 Appendix. Furthermore, in-text citations were also amended. |

**Reviewer 1:**

|  | **Comments** | **Response to comments by authors** |
| --- | --- | --- |
| 1 | The Author has tried to identify the correlation between ARGs detected and the antibiotic residues in the drinking water facilities. However, it is not clear why only a few antibiotic resistance genes were selected. | The author tried to correlate bacterial communities with physicochemical parameters, ARGs and antimicrobial residues. Initially, commonly prescribed antimicrobial agents and prevalent ARGs were included in the analysis. However, due to the failed optimization for screening ARGs and quantifying antimicrobial residues, only a few parameters were included in the current study (lines 467 to 471). |
| 2 | Were bacterial communities identified in the water common inhabitants of drinking water? | In this manuscript, drinking water is referred to as treated water. The bacterial composition of the drinking/treated water can be found in lines 298 to 317. |
| 3 | It looks like antibiotic residues were selected based on the ARGs identified in the water or vice versa. What is the relevance of this? | The antibiotics and ARGs selected were based on their prescription and prevalence in SA respectively (lines 467 to 471). |

**Reviewer 2:**

|  | **Comments** | **Response to comments by authors** |
| --- | --- | --- |
| 1 | Consider using “antimicrobial agents” instead when it comes to both antibiotics and fluconazole. | Thank you for the suggestion to use “antimicrobial agents”. However, the authors felt that the word “Antimicrobials” would fit better when referring to both antibiotics and fluconazole. |
| 2 | In Introduction justify the importance of monitoring fluconazole or antifungals because antibiotics are not the only antimicrobials quantified in this study. | The justification was provided in lines 61 to 64. |
| 3 | In Methods and materials most of the concentration units are represented as fractions (E.g.,ml/day), but concentration units of flow rate are not. Please represent all of these similarly. | The flow rate has been changed from mL min-1 to mL/min for consistency. |
| 4 | The title of each section is capitalized but is not the same for the Results section. Please use the same format. | The format was changed from “results” to “Results” |
| 5 | Finally, figures should have a good resolution in order to not lose text legibility. | Where applicable, images were replaced with higher-quality images. |
| 6 | Line 33-34-Please replace “In NW-E, antibiotic and fluconazole concentrations…” with “Regarding antimicrobial agents, antibiotic and fluconazole concentrations | Replaced as recommended |
| 7 | Line 63-It is appropriate to only use the term “human bodies” instead of “human and human bodies” in the same sentence. | It was changed to “human and animal bodies” |
| 8 | Line 79-Please use “antimicrobial agents ’residues” instead of “antibiotic residues because in the study fluconazole is also quantified. | It has been changed to “antimicrobial residues” rather than “antimicrobial agents’ residues” in the suggested line and others as well. |
| 9 | Line 95-Please rectify “environmental DNA (eDNA)” | It was changed to as recommended “environmental DNA (eDNA)” |
| 10 | Line 128-Verify the version of QIIME2 used in this study because the version of this software contains the year and the month. E.g., QIIME2 Version 2022.4 | The version of QIIME2 was changed to QIIME2 version 2022.2 (line 132) |
| 11 | Line 142-As a suggestion change “for screening for the presence…” by “for screening the presence…” | Changed as recommended (line 146). |
| 12 | Line 198-The slope symbol is “m”. Please rectify it. | The symbol is changed as recommended |
| 13 | Line 225-226-It is not clarified in which cases student’s t-test and ANOVA were used when data showed a normal distribution. | The error was fixed. The student’s t-test was the one used when data showed a normal distribution. ANOVA was deleted. |
| 14 | Line 274-275-Please detail the temperature results as was made with the other variables. The seasons in which temperature was measured should be specified in Table 2 and Table | Since this study was not based on seasonal variation, the statement was changed to “Temperature fluctuated between raw and treated water in NW-E and NW-C. In NW-E, the temperature range was between 10.3˚C and 24.1˚C. In NW-C, the range was between 13.6 ˚C and 20.9˚C. (Tables 2 and 3).” This description matches the ones of other variables. |
| 15 | Line 299-318-“Unclassified” group should not be mentioned as phyla or genera, so for example in the first case it can be reported that there were six phyla, detailing the proportion from each of them, but a specific percentage of the sequences have not taxonomy assignation. | “Unclassified” was removed as part of the phyla and genera |
| 16 | Line 491-492-Please cite the explanation of nitrite findings. | A citation was included [32] Amanatidou E, Samiotis G, Trikoilidou E, Pekridis G, Taousanidis N. Evaluating sedimentation problems in activated sludge treatment plants operating at complete sludge retention time. Water Research. 2015;69:20-9 |
| 17 | Line 679-686-An explanation about the risks of fluconazole presence in drinking water is recommended. | The explanation was provided “Its frequent application and recalcitrance to biodegradation in conventional wastewater treatment plants may lead to its prevalence in the water environment which may pose risks to water users” |
| 18 | Most of the manuscript is well-written in a comprehensive way; nonetheless, it is recommended that redaction could be improved for avoiding redundancy. | Reduced were applicable. |

In addition to the comments addressed in the tables above, editorial changes (typing errors) were also made in the revised manuscript document. We hope that you will find the revised manuscript satisfactory and are looking forward to your reply. Thank you for considering our paper for publication.

Yours sincerely,

Dr Karabo Tsholo

Research assistant: Unit for Environmental Science and Management (UESM)

North-West University

Potchefstroom
